# Healthcare workers’ behaviors on infection prevention and control and their determinants during the COVID-19 pandemic: a cross-sectional study based on the theoretical domains framework in Wuhan, China

**DOI:** 10.1186/s13690-021-00641-0

**Published:** 2021-06-30

**Authors:** Qiuxia Yang, Xuemei Wang, Qian Zhou, Li Tan, Xinping Zhang, Xiaoquan Lai

**Affiliations:** 1 School of Medicine and Health Management, Tongji Medical College, Huazhong University of Science and Technology, No. 13, Hangkong Road, Wuhan, 430030 Hubei Province China; 2 Tongji Hospital, Tongji Medical College, Huazhong University of Science and Technology, No. 1095, Jiefang Avenue, Wuhan, 430030 Hubei Province China

**Keywords:** COVID-19, Infection prevention and control, Hand hygiene, Personal protective equipment, Healthcare workers, Theoretical domains framework

## Abstract

**Background:**

Infection prevention and control (IPC) measures are crucial to combat the COVID-19 pandemic. This study aimed to explore the levels and determinants of HCWs’ IPC behaviors based on the theoretical domains framework (TDF), which has been shown to be effective in guiding behavior change.

**Methods:**

A cross-sectional survey was conducted in Wuhan, China in January 2020. Self-reported hand hygiene and droplet isolation behaviors (including the use of masks, gloves, goggles and gowns) were set as dependent variables. TDF domains and HCWs’ characteristics were independent variables. Negative binomial regression analyses were performed to explore their relationships.

**Results:**

HCWs reported good IPC behaviors, while the compliance with goggle and gown use was relatively low (below 85%). Environmental context and resources domain was significantly related to hand hygiene (β = 0.018, *p* = 0.026), overall droplet isolation behaviors (β = 0.056, *p* = 0.001), goggle (β = 0.098, *p* = 0.001) and gown use (β = 0.101. *p* < 0.001). Knowledge domain was significantly related to goggle (β = 0.081, *p* = 0.005) and gown use (β = 0.053, *p* = 0.013). Emotion domain was a predictor of overall droplet isolation behaviors (β = 0.043, *p* = 0.016), goggle (β = 0.074, *p* = 0.026) and gown use (β = 0.106, *p* < 0.001). Social influences domain was a predictor of overall droplet isolation behaviors (β = 0.031, *p* = 0.029) and gown use (β = 0.039, *p* = 0.035). HCWs in high-risk departments had better behaviors of gown use (β = 0.158, *p* = 0.032). HCWs who had encountered confirmed or suspected patients reported worse behaviors of goggle (β = − 0.127, *p* = 0.050) and gown use (β = − 0.153, *p* = 0.003).

**Conclusions:**

Adequate personal protective materials and human resources, education and training, as well as supervision and role model setting are necessary to improve IPC behaviors regarding the COVID-19 pandemic.

**Supplementary Information:**

The online version contains supplementary material available at 10.1186/s13690-021-00641-0.

## Background

Since the first public reporting of COVID-19 on 31 December 2019, Wuhan city in China has become the focus of global attention. With the development of the epidemic, human-to-human transmission of severe acute respiratory syndrome coronavirus 2 (SARS-CoV-2) was eventually confirmed [1]. Approximately 5 million people left Wuhan during the Spring Festival in 2020, leading to rapid spread of SARS-CoV-2 all over China [2]. The increasing spread speed was so alarming that a constitution of Public Health Emergency of International Concern was officially declared [3]. Meanwhile, SARS-CoV-2 was also found in other countries, which caused a global pandemic in the following months. The ongoing COVID-19 pandemic has caused nearly 171million confirmed cases and claimed more than 3.6million lives worldwide as of 3 June 2020 [4].

Infection prevention and control (IPC) measures are important to prevent the spread of infection caused by SARS-CoV-2 [5]. The two main transmission routes of SARS-CoV-2 are droplet and contact transmission [6]. When patients infected with COVID-19 cough or exhale, respiratory droplets containing SARS-CoV-2 are produced, and anyone close to them can inhale these droplets and become infected [6]. SARS-CoV-2 can also survive on environmental surfaces for 4–72 h [7] and can be transmitted through direct physical contact and indirect contact via contaminated environmental surfaces or materials [6, 8]. As a crucial source of infections, hospitals should avoid becoming a vehicle for transmission to patients and front-line healthcare workers (HCWs) when dealing with suspected or confirmed cases [6]. Therefore, HCWs should implement appropriate IPC behaviors including personal protective equipment (PPE) use and hand hygiene, to protect patients and themselves from infection.

However, as illustrated by low compliance in previous studies, HCWs are having difficulty in complying to IPC measures in practice [6, 9]. Therefore, authorities and hospitals need to consider how to support HCWs to implement these measures [10]. Many studies have attempted to explore factors influencing hand hygiene to help develop evidence-based interventions [10–13]; among which knowledge, attitudes and socio-demographic characteristic are the most frequently explored factors [14, 15]. In addition, hand hygiene facilities [16], workload [16], self-efficacy [17, 18], and social influences [15] are often regarded as factors associated with hand hygiene. A review study found that hand hygiene facilities and workload may be the main factors associated with poor hand hygiene compliance in developing countries or regions [16]. For PPE use, only few studies explored relevant factors, such as workload [19], attitudes [19], beliefs [20] and risk perception [21]. During the COVID-19 pandemic, resource shortages are prominent. Facilities and workload may be the most important factors affecting hand hygiene and PPE use, which requires more research. Besides, previous studies often examined limited factors only, and few of them have a theoretical basis [22]. The HCWs’ behaviors of hand hygiene and PPE use are affected by abroad range of factors [10, 22]. The understanding of HCWs’ IPC behaviors and their determinants remains limited [23].

The determinants of HCWs’ IPC behaviors can be identified using psychological frameworks of behavior change, which are promising tools for understanding and improving hand hygiene practice [22]. Only one study assessed the determinants of hand hygiene by using health action process approach theory during the COVID-19 pandemic; in which, self-efficacy and coping self-efficacy were found to be significantly associated with hand hygiene adherence [17]. Nevertheless, this study examined few social–cognitive factors.

The Theoretical Domains Framework (TDF) was adopted in our study for research design and data interpretation, which could fill the research gap from a comprehensive perspective. As a well-validated, consensus-based, and integrative theoretical framework, TDF can promote the understanding of HCWs’ behaviors, such as IPC practice, by examining potential underlying factors [11, 24–26]. TDF mainly consists of 12 domains developed from 33 theories, covering knowledge, skills, social/professional role and identity, beliefs about capabilities, beliefs about consequences, goals, memory and attention, environmental context and resources, social influences, emotion, behavioral regulation, and nature of behavior [25, 26]. TDF covers almost all the factors frequently explored in previous studies, including facilities and workload. As such, TDF should be applied to identify determinants of HCWs’ IPC behaviors during the COVID-19 pandemic to develop targeted strategies for optimizing such behaviors at this critical time [22, 26].

This study aims to explore the levels and determinants of HCWs’ IPC behaviors based on TDF in Wuhan, China to provide data for the prevention and control of the COVID-19 pandemic or other possible future epidemics. And the following research hypotheses are proposed:

- H1: HCWs have better IPC behaviors after the COVID-19 outbreak than before.
- H2: TDF domains are associated with HCWs’ IPC behaviors.

## Methods

### Setting

This study was conducted in a well-known tertiary public hospital in Wuhan city, Hubei province in January 2020. Wuhan is located in central China, has a resident population of 11.08 million, and is considered a middle-range economic development area of China. The surveyed hospital is a traditional teaching hospital with more than 6000 inpatient beds and provides over 250, 000 inpatient and 300,000 outpatient services per year.

### Participants and data collection

HCWs in the selected hospital who were willing to participate were invited to fill in the questionnaire. Medical technicians and hospital administrators were excluded from this study. A structured anonymous questionnaire was used to collect data on self-reported IPC behaviors, TDF domains, and HCWs’ characteristics. Written informed consent was obtained before each respondent filled in the questionnaire. Our trained investigators collected the completed questionnaires. HCWs were encouraged to check the questionnaires and fill in the missing items if they exist. The survey took about 15 min on average.

### Measurements

#### Self-reported IPC behaviors

According to the guideline proposed by world health organization (WHO) [5], nine items were developed to capture HCWs’ compliance with the recommended IPC measures. Five items were related to hand hygiene compliance (before patient contact, before aseptic procedures, after body fluid exposure, after patient contact, and after contact with patient surroundings). Four items were related to droplet isolation behaviors, including use of masks, gloves, goggles, and gowns.

For each item, the participant reported the times that he/she complied with the recommended IPC guidelines in the 10 corresponding behaviors (0–10 times) [27]. For example, how many times did you perform hand hygiene in ten times before patient contact? HCWs answered the same items twice – once retrospectively for the time 1 month before the COVID-19 outbreak and once for 1 month after the outbreak to observe the change of IPC behaviors with the outbreak. We chose self-reported behavior rather than direct observation to prevent observers from being at the risk of infection. IPC behavior compliance is equal to the number of self-reported behaviors conforming to guidelines/total number of all possible behaviors.

#### TDF domains

The TDF was used to develop the questionnaire, analyze data, and interpret results from the survey [24, 25]. Initially, 79 items belonging to 10 domains of TDF were generated based on the results of previous qualitative studies related to hand hygiene [11–13, 26, 28]. The 10 domains are knowledge, skills, beliefs about capabilities, beliefs about consequences, memory and attention, environmental context and resources, social/professional role and identity, social influences, goals, and emotion. The behavioral regulation and nature of behavior domains were not included in this study due to the following reasons. First, they were not often identified in previous qualitative studies on hand hygiene based on TDF [11–13, 28, 29]. Second, behavioral regulation domain is more like the direction of action than a factor of behaviors [29]. Similarly, the nature of behaviors is not a determinant but rather a set of characteristics (e.g., frequent or one-off, approach, or avoid) that can be used to describe behaviors [13].

Items with the same or similar meaning were merged, and 45 items were obtained. Meanwhile, each item from the English version was translated into Chinese. A focus group discussion was held to put forward suggestions on the necessity and appropriateness of domain attribution and the accuracy of translation for each item. The focus group members were graduate students and professors engaged in IPC research. According to the suggestions, five items were deleted and the 40 remaining items were revised.

Before the formal survey, a 40-item questionnaire was distributed to six experts in the field of IPC to assess the suitability of the items. According to their suggestions, one item of beliefs about capabilities, two items of beliefs about consequences, and one item of goals were deleted, while one item of knowledge was added. Finally, a 37-item TDF instrument of hand hygiene behavior was obtained.

A 39-item TDF instrument for droplet isolation was constructed referring to the TDF instrument for hand hygiene and the WHO IPC guideline [5] because they are both personal protective behavior and no study on droplet isolation behavior was conducted based on TDF previously. Each item of the TDF instrument for droplet isolation was also discussed and revised through focused group discussions.

All items were rated on a five-point Likert scale, ranging from “strongly agree” (5) to “strongly disagree” (1) or from “know completely” (5) to “not know at all” (1). All reverse items were handled positively when analyzing data.

#### HCWs’ characteristics

Two professional risk factors (contact with confirmed or suspected patients, working in high-risk departments) were included as independent variables. The departments that are at high risk of admitting COVID-19 patients were coded into high-risk departments, including respiratory medicine, infectious disease, emergency, and general intensive care unit. The respondents were asked whether they were exposed to confirmed and suspected patients (defined as flu-like cases with a body temperature above 38 °C and sore throat or cough). Each item was scored 1 if the answer was yes and 0 if the answer was no. In addition, HCWs’ demographic characteristics (e.g., gender, occupation, age, working years, education degree, title) were investigated.

### Statistical analyses

All the analyses were performed using Stata 15.0 (Stata Corp LP, College Station, TX, USA). Descriptive statistics were applied to describe self-reported IPC behaviors, TDF domains, and HCWs’ characteristics. Mann-Whitney U test was used to compare HCWs’ IPC behaviors before and after the pandemic. Confirmatory factor analyses (CFA) were conducted to validate TDF instruments, and items with a factor loading above 0.40 were reserved [30]. Internal consistency reliability was estimated by Cronbach’s alpha (Cronbach’s alpha > 0.7, acceptable; > 0.6, questionable) [31]. One item would be deleted if the Cronbach’s alpha of the targeted domain increased when the item was deleted. For the variables of HCWs’ characteristics, gender and occupation were treated as categorical variables, and age and work years were used as continuous variables. LR/ BIC/ AIC tests were performed to determine the ordinal variables (educational level, title) as continuous or categorical variables [32]. Negative binomial regression analyses were performed to explore the determinants of IPC behaviors. The significance level was set at *p ≤* 0.05. Stratified analyses were used to check the robustness of self-reported behaviors, and the data were divided by gender, occupation, and age of HCWs.

## Results

A total of 853 HCWs were surveyed, and 768 (90.0%) returned a valid questionnaire. The average age and working years of HCWs were 30.99 ± 6.53 and 7.56 ± 6.71, respectively. The details of HCWs’ characteristics are shown in Table 1.

**Table 1:** Characteristics of the study participants in Wuhan China, 2019 (*N* = 768)

	n (%)
Gender	
Male	153(19.9)
Female	614(80.1)
Educational level	
Associate degree or below	18(2.4)
Bachelor’s degree	539(70.5)
Master’s degree	74(9.7)
Doctor’s degree	134(17.5)
Occupation	
Physician	252(33.1)
Nurse	510(66.9)
Technical title	
To be appraised	100(13.6)
Junior	361(49.0)
Middle	229(31.1)
Associate senior	39(5.3)
Senior	8(1.1)
Contact with confirmed or suspected patients	
Yes	95(15.1)
No	536(84.9)
High-risk departments	
Yes	47(6.1)
No	721(93.9)

HCWs reported better IPC behaviors during the COVID-19 pandemic compared with before the outbreak. The compliance of five moments of hand hygiene ranged from 93.97 to 99.23%. The compliance of mask and glove use was above 95%, whereas goggle and gown use was above 80% but less than 90% (Table 2).

**Table 2:** The levels of self-reported behaviors on infection prevention and control among healthcare workers in Wuhan China, 2019

	Compliance before the outbreak (%)	Compliance during the outbreak (%)	*Z*
Overall hand hygiene compliance	88.69	96.37	11.746^*^
Before patient contact	82.80	94.21	10.923^*^
Before aseptic procedures	95.04	98.50	6.749^*^
After body fluid exposure	96.25	99.23	7.076^*^
After patient contact	90.75	97.57	9.528^*^
After touching patient surroundings	84.53	93.97	9.852^*^
Overall droplet isolation compliance	76.93	87.94	9.127^*^
Use of mask	93.41	97.94	7.587^*^
Use of glove	85.81	95.82	10.021^*^
Use of goggle	68.51	81.82	8.075^*^
Use of gown	73.98	85.52	7.932^*^

^*^ *p* < 0.001

Two items of TDF instrument for hand hygiene were deleted according to the results of CFA and internal consistency reliability analyses. The factor loadings for the retained items were above 0.4, ranging from 0.460 to 0.873 and 0.493 to 0.938 for hand hygiene and droplet isolation respectively. The detailed factor loadings for each item are presented in Additional file 2:Table S1 and Table S2.

The results of descriptive and reliability analyses of TDF domains are shown in Table 3. The mean scores of TDF domains for hand hygiene ranged from 4.12 to 4.87, while the mean scores of TDF domains for droplet isolation ranged from 4.13 to 4.82. The Cronbach’s α of most TDF domains was above 0.7, whereas the Cronbach’s α of memory and attention, beliefs about consequences (only for hand hygiene), and social influences (only for droplet isolation) was less than 0.7 (0.619, 0.668, 0.602, 0.623, respectively).

**Table 3:** The mean score and Cronbach’s α of TDF domains reported by healthcare workers in Wuhan China, 2019

Domains	Hand hygiene		Droplet isolation	
	Mean (SD)	α	Mean (SD)	α
Knowledge	4.87(0.42)	0.768	4.683(0.67)	0.819
Skills	4.76(0.50)	0.792	4.515(0.70)	0.812
Memory and attention	4.12(1.33)	0.619	4.144(1.26)	0.602
Environmental context and resources	4.26(1.20)	0.763	4.137(1.23)	0.790
Social influences	4.62(0.78)	0.723	4.292(1.17)	0.623
Beliefs about consequences	4.83(0.51)	0.668	4.820(0.50)	0.753
Beliefs about capabilities	4.74(0.55)	0.864	4.519(0.78)	0.932
Social/professional role and identity	4.79(0.54)	0.846	4.792(0.52)	0.882
Goals	4.84(0.46)	0.835	4.822(0.47)	0.839
Emotion	4.74(0.58)	0.810	4.73(0.59)	0.855

*Note. TDF* Theoretical Domains Framework; *SD* Standard Deviation

All the significant variables in the negative binomial regression models are shown in Table 4. As the LR/ BIC/AIC tests in all models were examined with *p* > 0.05, the ordinal variables (education level, title) were included as continuous variables. Environmental context and resources domain was a significant determinant of overall hand hygiene compliance (β = 0.018, *p* = 0.026), overall droplet isolation behaviors (β = 0.056, *p* = 0.001), goggle use (β = 0.098, *p* = 0.001), and gown use (β = 0.101, *p* < 0.001). Knowledge domain was significantly related to goggle use (β = 0.081, *p* = 0.005) and gown (β = 0.053, *p* = 0.013). Emotion domain significantly influenced the overall droplet isolation behaviors (β = 0.043, *p* = 0.016), goggle (β = 0.074, *p* = 0.026) and gown use (β = 0.106, *p* < 0.001). Social influences domain significantly influenced the overall droplet isolation behaviors (β = 0.031, *p* = 0.029) and use of gown (β = 0.039, *p* = 0.035).

**Table 4:** Determinants of behaviors on infection prevention and control among healthcare workers in Wuhan China, 2019

	Nonstandardized Coefficients		Standardized coefficients	*p*
	b	SD	β	
**Overall hand hygiene compliance**				
Environmental context and resources	0.022	0.010	0.018	0.026
**Compliance of overall droplet isolation behaviors**				
Environmental context and resources	0.075	0.023	0.056	0.001
Social influences	0.051	0.023	0.031	0.029
Emotion	0.077	0.032	0.043	0.016
**Use of goggle**				
Contact with confirmed or suspected patients	−0.127	0.065	−0.127	0.050
Knowledge	0.174	0.062	0.081	0.005
Environmental context and resources	0.133	0.040	0.098	0.001
Emotion	0.134	0.060	0.074	0.026
**Use of gown**				
Contact with confirmed or suspected patients	−0.152	0.051	− 0.153	0.003
Working in high-risk departments	0.158	0.074	0.158	0.032
Knowledge	0.114	0.046	0.053	0.013
Environmental context and resources	0.137	0.031	0.101	< 0.001
Social influences	0.064	0.030	0.039	0.035
Emotion	0.193	0.048	0.106	< 0.001
Education degree	−0.080	0.035	−0.065	0.021

Note. *SD* Standard Deviation; *Educational level* ‘associate degree or below’ was coded as 1, ‘bachelor’s degree’ was coded as 2, ‘master’s degree’ was coded as 3, ‘doctor’s degree’ was coded as 4

HCWs in high-risk departments showed better behavior of gown use (β = 0.158, *p* = 0.032), while HCWs who had contact with confirmed or suspected patients had worse behaviors of goggle (β = − 0.127, *p* = 0.050) and gown use (β = − 0.153, *p* = 0.003). HCWs with higher education degree reported low compliance of gown use (β = − 0.080, *p* = 0.003). No significant variables were included in the regression models of mask and glove use. The results of the stratified analysis are robust and consistent with the main results (Additional file 3: Table S3).

## Discussion

In general, HCWs reported better IPC behaviors during the COVID-19 pandemic than before, with relatively high compliance of hand hygiene, mask use, and glove use and relatively low compliance of goggle and gown use. The negative binomial regression analyses showed that the environmental context and resources domain was significantly associated with overall hand hygiene compliance, overall droplet isolation behaviors, goggle use, and gown use. Knowledge domain was significantly associated with goggle and gown use. Emotion domain was a significant predictor of overall droplet isolation behaviors, goggle use, and gown use. Social influences domain was a significant predictor of overall droplet isolation behaviors and gown use. HCWs who worked in high-risk departments showed better behavior of gown use, while HCWs who had contact with confirmed or suspected patients reported worse behaviors of goggle and gown use.

The higher compliance of IPC behaviors during the COVID-19 pandemic indicated that HCWs improved their IPC behaviors after the outbreak, as indicated in previous studies [17, 27]. The selected hospital is close to the seafood and live animal market, the first detected outbreak site of COVID-19 in China, and has become one of the worst affected hospitals during the outbreak. Pamphlets on COVID-19 prevention and control were distributed to HCWs in every clinical ward at the early stage of the outbreak. It may be that HCWs had a strong sense of IPC, so they generally showed good IPC behaviors. However, HCWs’ relatively low compliance of goggle and gown use (below 86%) may make them highly susceptible to SARS-CoV-2 infection and transmission.

In this study, the environmental context and resources domain was identified as a significant determinant of hand hygiene behaviors, consistent with previous studies [13, 16]. Hence, the lack of hand hygiene facilities and products may be the most critical obstacles to HCWs’ hand hygiene behaviors during the COVID-19 pandemic. As mentioned in previous studies [16, 33], heavy workload was also a critical barrier to hand hygiene, which was often linked to the shortage of human resources especially in developing countries [34]. The COVID-19 pandemic has made such shortage more serious. A qualitative review also concluded that hand hygiene facilities and workload were important determinants of HCWs’ adherence to IPC guidelines [10]. In a life-threatening emergency, resources may be the most crucial constraints because HCWs have strong motivation to get rid of other obstacles and perform hand hygiene. In addition, some results of this study are inconsistent with previous reports. For example, beliefs about capabilities, memory and attention, and social influences domains were identified as influencing factors in previous studies, whereas their relationships with hand hygiene showed no significance in the present study [13, 18, 28]. The self-reported compliance possibly displayed limited variance so the regression analysis cannot extract sufficient information to detect the relationships. Moreover, these differences may be due to differences in healthcare facility settings or cross-cultural differences.

Environmental context and resources domain was also significantly associated with overall droplet isolation behaviors, goggle use, and gown use. During the epidemic, the demand and consumption of PPE are huge. HCWs cannot fully comply with the guidelines when PPE stocks are insufficient in the hospitals. In January 2020, hospitals in Wuhan of Hubei province faced a pronounced lack of PPE supplies at the initial stage, mainly gowns and goggles, and eight hospitals even issued announcements successively to collect PPE from the public [35]. Similar to hand hygiene, in addition to material supply, increasing human resources in the affected hospitals are critical to improve compliance with droplet isolation behaviors, especially goggle and gown use. As of January 282,020, nearly 6000 HCWs from all over China came to support Hubei province, and more than 10,000 beds were provided in Wuhan [36]. Adequate HCW staffing is considered a core component of effective IPC programs by the WHO [37].

Lack of knowledge was found to be associated with lower compliance of goggle and gown use among HCWs. Hand hygiene as well as glove and mask use are easy and common to conduct, but many HCWs may not know when and how to use goggle and gown correctly [38]. One possible explanation is that HCWs have less experience in training and practice regarding the use of goggle and gown because they are only used in certain medical situations such as controlling the SARS epidemic [39]. Therefore, training regarding goggle and gown use should be strengthened, which was also suggested by a previous study that reported that training could improve facial protective equipment use [40]. Hence, emotions may prompt medical staff to hand hygiene practices.

Emotion domain was significantly associated with the overall droplet isolation behaviors, goggle use, and gown use. This revealed that emotion including guilt, shame, and fear caused by not following the guidelines may prompt HCWs to implement droplet isolation behaviors. Emotion can also cause mental tension and stress, whereas most HCWs are more likely to turn their stress into motivation for action, which may be verified by their high compliance with IPC guidelines during the epidemic. However, a prior study suggested that too much fear may cause avoidance and inattentiveness [41]. More precisely, HCWs might be reminded of the COVID-19 pandemic when seeing their colleagues in goggles or gowns, which might become a reminder of pressure that some HCWs may want to avoid. Meanwhile, HCWs were required to follow droplet isolation guidelines, which may cause constant struggles and high emotional burdens for HCWs. Therefore, education and training on the value of PPE in infection control especially self-protection, as well as communication and counselling on the emotional burden, should be strengthened to promote a positive effect of emotion on HCWs’ behavior change.

In particular, social influences domain was significantly associated with the overall droplet isolation behaviors and gown use, indicating that external pressure (from colleagues, department leaders, infection management personnel, etc.) could improve HCWs’ overall droplet isolation behaviors and gown use. Social influences domain was not a significant predictor of goggle use, and the possible reason may be that goggles are less visible than a gown. HCWs are easier to be reminded by colleagues in gowns, which are often used in severe cases, such as SARS and the COVID-19 pandemic [5, 38]. Meanwhile, inconsistent with previous studies [13], social influences domain was not a determinant of hand hygiene, mask use, and glove use, which may be explained by their high compliance among most HCWs during the COVID-19 pandemic. Thus, supervision and role model setting in the clinical departments may be practical strategies to improve gown use.

HCWs in high-risk departments had a better behavior pattern of gown use, probably because they received more training on PPE use. In addition, HCWs in high-risk departments were more likely to have a stronger sense of self-protection and infection prevention and control because of higher infection incidence in their areas [27]. In Contrast expectations, HCWs who had encountered confirmed or suspected patients had poor behaviors of goggle and gown use. The reason may be that the presence of confirmed or suspected cases exacerbated the PPE shortage and increased the already high workload of HCWs in China [27, 33, 35, 36]. Furthermore, previous studies have demonstrated that PPE use can increase HCWs’ workload [10]. As we know, the procedures of gown and goggle use are more complex and time consuming [39].

None of the TDF domains were significantly associated with mask and glove use, which may because self-reported mask and glove use of HCWs’ in the sample were too high to display sufficient variance to identify the significant TDF domains. Additionally, the procedures of mask and glove use are simple, so it is easy for HCWs to adhere to the guidelines in practice [5, 39]. With the outbreak, HCWs were more willing to spend effort to use masks and gloves to protect themselves and their patients, and the compliance rate of mask and glove use reached above 95%. No shortage of masks and gloves occurred at the early stage of the COVID-19 outbreak. However, with the development of the epidemic, the lack of PPE, including masks and gloves, has gradually become a major problem worldwide, which may hinder compliance with mask and glove use [42].

This study has some limitations. First, HCWs’ self-reported IPC behaviors may be overestimated due to social desirability or self-serving bias, which may lead to low variance in the sample [15]. Given that participants were assured that their responses would remain strictly confidential, we think that the bias has been minimized [15]. In addition, data collection was retrospective and probably susceptible to memory effects. Due to the lack of high-cost technical measures such as video cameras (which are often regarded as intrusive by HCWs) [43], directly observing actual IPC behaviors of each HCW during the pandemic is difficult. Although self-reported hand hygiene was not the golden standard [44], a study concluded that self-reported behavior among HCWs was comparable to behavior measured by direct observation [45]. Moreover, self-report method was often used to measure HCWs’ behaviors [13, 20, 46], which also made it possible to assess the levels and determinants of IPC behaviors in practice on a large scale [13]. Second, given that this was a cross-sectional study, the causal relationships between TDF domains and HCWs’ IPC behaviors should be explained with caution. Third, the Cronbach’s alpha of memory and attention, beliefs about consequences and social influences were questionable, probably because these domains have measured inconsistent and broad constructs, which needs to be improved in the future.

## Conclusions

During the COVID-19 pandemic, self-reported IPC behaviors among HCWs were better than before, whereas the compliance of goggle and gown use was not very satisfactory. Environment context and resources, knowledge, emotion, and social influences were identified as determinants of IPC behaviors. HCWs in high-risk departments had better behaviors of gown use, whereas HCWs who had encountered confirmed or suspected patients reported worse behaviors of goggle and gown use. To improve HCWs’ IPC behaviors in response to the COVID-19 pandemic or possible future epidemics, policy makers and authorities should invest in education and create employment opportunities for HCWs to improve global health security in the long term [34]. Increasing health human resources will reduce HCWs’ workload, thereby improving IPC behaviors. For healthcare facilities, adequate personal protective materials, education and training, supervision and role model setting may be essential. For HCWs, it is important to constantly learn knowledge and implement the IPC guidelines. In the future, longitudinal, high-quality research on IPC behaviors is needed. Further studies are warranted to explore the determinants of IPC behaviors in different regions or stages of COVID-19. The specific reasons for the noncompliance with IPC guidelines of HCWs who have poor IPC behaviors should be differentiated in the future. Further research is also needed to improve the reliability and validity of the TDF instrument.

## Supplementary Information

**Additional file 1:.** Survey of infection prevention and control behaviors among healthcare workers.

**Additional file 2: Table S1.** TDF items for hand hygiene and their factor loadings; **Table S2.** TDF items for droplet isolation and their factor loadings.

**Additional file 3: Table S3.** Robustness check.

## Data Availability

The dataset generated and analyzed during the current study is available from the corresponding author on reasonable request.

## Abbreviations

COVID-19: Coronavirus disease 2019
SARS-CoV-2: Severe acute respiratory syndrome coronavirus 2
IPC: Infection prevention and control
HCWs: Healthcare workers
PPE: Personal protective equipment
TDF: Theoretical domains framework
WHO: World health organization
