# Supplementary material for: Healthcare workers’ behaviors on infection prevention and control and their determinants during the COVID-19 pandemic: a cross-sectional study based on the theoretical domains framework in Wuhan, China
Source: Arch Public Health. 2021 Jun 30;79:118. doi: 10.1186/s13690-021-00641-0 (PMC8242273; doi:10.1186/s13690-021-00641-0)
Supplement: Supplementary file 1 — Additional file 1:. Survey of infection prevention and control behaviors among healthcare workers. [file 13690_2021_641_MOESM1_ESM.docx]

**Survey of infection prevention and control behaviors among healthcare workers**

**Dear physicians/nurses,**

Greeting!

We are conducting a survey of healthcare workers’ infection prevention and control behaviors regarding the COVID-19 pandemic. We invited you to complete this 15-minute questionnaire and your responses are of great values to help improve infection prevention and control practice in hospitals. Your personal information would be confidential, and the responses were only used for academic research only. Thanks very much for your participation.

School of Medicine and Health Management

Tongji Medical College, Huazhong University of Science and Technology

The suspected (high-risk) patient: Influenza-like cases, fever ≥38℃, accompanied by a sore throat or cough.

1. Did you treat/care the confirmed or suspected the COVID-19 patient?

□yes ; □no

2.Your department:

**How many times have you done hand hygiene in the 10 times before you contacted the patient?**

**Part 1: Infection prevention and control behaviors**

Note: this part of the questions **does not have the correct answer**, please fill in according to your actual practice.

|  | **One recent month**  **（after the COVID-19 outbreak）** | | | | | | | | | | |
| --- | --- | --- | --- | --- | --- | --- | --- | --- | --- | --- | --- |
|  | 10 | 9 | 8 | 7 | 6 | 5 | 4 | 3 | 2 | 1 | 0 |
| 1. How many times did you perform hand hygiene in the ten times before patient contact? | □ | □ | □ | □ | □ | □ | □ | □ | □ | □ | □ |
| 2. How many times did you perform hand hygiene in the ten times before aseptic procedures? | □ | □ | □ | □ | □ | □ | □ | □ | □ | □ | □ |
| 3. How many times did you perform hand hygiene in the ten times after patients’ body fluid exposure? | □ | □ | □ | □ | □ | □ | □ | □ | □ | □ | □ |
| 4. How many times did you perform hand hygiene in the ten times after patient contact? | □ | □ | □ | □ | □ | □ | □ | □ | □ | □ | □ |
| 5. How many times did you perform hand hygiene in the ten times after contact with patient surroundings? | □ | □ | □ | □ | □ | □ | □ | □ | □ | □ | □ |
| 6. In the ten times of contact with confirmed or suspected cases, how many times did you wear a mask? | □ | □ | □ | □ | □ | □ | □ | □ | □ | □ | □ |
| 7. In the ten times of contact with confirmed or suspected cases, how many times did you wear a glove? | □ | □ | □ | □ | □ | □ | □ | □ | □ | □ | □ |
| 8. In the ten times of contact with confirmed or suspected cases, how many times did you wear a goggle? | □ | □ | □ | □ | □ | □ | □ | □ | □ | □ | □ |
| 9. In the ten times of contact with confirmed or suspected cases, how many times did you wear a gown? | □ | □ | □ | □ | □ | □ | □ | □ | □ | □ | □ |

|  | **One month before**  **（before the COVID-19 outbreak）** | | | | | | | | | | |
| --- | --- | --- | --- | --- | --- | --- | --- | --- | --- | --- | --- |
|  | 10 | 9 | 8 | 7 | 6 | 5 | 4 | 3 | 2 | 1 | 0 |
| 1. How many times did you perform hand hygiene in the ten times before patient contact? | □ | □ | □ | □ | □ | □ | □ | □ | □ | □ | □ |
| 2. How many times did you perform hand hygiene in the ten times before aseptic procedures? | □ | □ | □ | □ | □ | □ | □ | □ | □ | □ | □ |
| 3. How many times did you perform hand hygiene in the ten times after patients’ body fluid exposure? | □ | □ | □ | □ | □ | □ | □ | □ | □ | □ | □ |
| 4. How many times did you perform hand hygiene in the ten times after patient contact? | □ | □ | □ | □ | □ | □ | □ | □ | □ | □ | □ |
| 5. How many times did you perform hand hygiene in the ten times after contact with patient surroundings? | □ | □ | □ | □ | □ | □ | □ | □ | □ | □ | □ |
| 6. In the ten times of contact with suspected cases, how many times did you wear a mask? | □ | □ | □ | □ | □ | □ | □ | □ | □ | □ | □ |
| 7. In the ten times of contact with suspected cases, how many times did you wear a glove? | □ | □ | □ | □ | □ | □ | □ | □ | □ | □ | □ |
| 8. In the ten times of contact with suspected cases, how many times did you wear a goggle? | □ | □ | □ | □ | □ | □ | □ | □ | □ | □ | □ |
| 9. In the ten times of contact with suspected cases, how many times did you wear a gown? | □ | □ | □ | □ | □ | □ | □ | □ | □ | □ | □ |

**Part 2: Factors related to hand hygiene**

**Note:** this part of the questions **does not have the correct answer**, please fill in according to your actual situation.

|  | Strongly agree | Agree | neutral | Disagree | Strongly disagree |
| --- | --- | --- | --- | --- | --- |
| 1.I am aware of the five moments of hand hygiene | 🗆 | 🗆 | 🗆 | 🗆 | 🗆 |
| 2. I know the content of the current hand hygiene guidelines | 🗆 | 🗆 | 🗆 | 🗆 | 🗆 |
| 3.I am aware of how to perform six-step hand washing technique | 🗆 | 🗆 | 🗆 | 🗆 | 🗆 |
| 4.I am aware of evidence linking hand hygiene to healthcare-associated infections | 🗆 | 🗆 | 🗆 | 🗆 | 🗆 |
| 5.I often participate in hand hygiene training | 🗆 | 🗆 | 🗆 | 🗆 | 🗆 |
| 6.Hand hygiene is an important part of my professional training | 🗆 | 🗆 | 🗆 | 🗆 | 🗆 |
| 7.Practicing hand hygiene is a habit of me | 🗆 | 🗆 | 🗆 | 🗆 | 🗆 |
| 8.Easily visible hand hygiene stations make it easier to remember to practice hand hygiene | 🗆 | 🗆 | 🗆 | 🗆 | 🗆 |
| 9.I find myself forgetting to practice hand hygiene more often than others | 🗆 | 🗆 | 🗆 | 🗆 | 🗆 |
| 10.I sometimes forget to do hand hygiene because I get distracted by other things | 🗆 | 🗆 | 🗆 | 🗆 | 🗆 |
| 11.The communication with colleagues can promote me to perform hand hygiene | 🗆 | 🗆 | 🗆 | 🗆 | 🗆 |
| 12.I am often too busy to do hand hygiene | 🗆 | 🗆 | 🗆 | 🗆 | 🗆 |
| 13. Hand hygiene facilities are adequate | 🗆 | 🗆 | 🗆 | 🗆 | 🗆 |
| 14.I can easily access to hand hygiene facilities | 🗆 | 🗆 | 🗆 | 🗆 | 🗆 |
| 15.When busy, I am less likely to comply with hand hygiene guidelines | 🗆 | 🗆 | 🗆 | 🗆 | 🗆 |
| 16.The type of my job makes it difficult to do hand hygiene | 🗆 | 🗆 | 🗆 | 🗆 | 🗆 |
| 17.My colleagues do hand hygiene as often as they should | 🗆 | 🗆 | 🗆 | 🗆 | 🗆 |
| 18.Department leaders attach importance to hand hygiene | 🗆 | 🗆 | 🗆 | 🗆 | 🗆 |
| 19.I do hand hygiene because my patients expect me to do hand hygiene | 🗆 | 🗆 | 🗆 | 🗆 | 🗆 |
| 20.The infection management department/Infection prevention and control team urges me to do hand hygiene | 🗆 | 🗆 | 🗆 | 🗆 | 🗆 |
| 21.Staffs who do well in hand hygiene will be praised and encouraged | 🗆 | 🗆 | 🗆 | 🗆 | 🗆 |
| 22.I consistently do hand hygiene because my colleagues do so | 🗆 | 🗆 | 🗆 | 🗆 | 🗆 |
| 23.Hand hygiene reduces/prevents the transmission of infection | 🗆 | 🗆 | 🗆 | 🗆 | 🗆 |
| 24.Hand hygiene protects me from infection | 🗆 | 🗆 | 🗆 | 🗆 | 🗆 |
| 25.Hand hygiene procedure is easy to practice | 🗆 | 🗆 | 🗆 | 🗆 | 🗆 |
| 26.I am confident in my ability to comply with hand hygiene guidelines | 🗆 | 🗆 | 🗆 | 🗆 | 🗆 |
| 27.I can do hand hygiene as frequently as guidelines required | 🗆 | 🗆 | 🗆 | 🗆 | 🗆 |
| 28.I do hand hygiene to protect my patients | 🗆 | 🗆 | 🗆 | 🗆 | 🗆 |
| 29.I do hand hygiene to protect my family | 🗆 | 🗆 | 🗆 | 🗆 | 🗆 |
| 30. Hand hygiene is something I must do as part of my role | 🗆 | 🗆 | 🗆 | 🗆 | 🗆 |
| 31.By regularly doing hand hygiene, I can be a role model for others | 🗆 | 🗆 | 🗆 | 🗆 | 🗆 |
| 32.Hand hygiene is always a necessity | 🗆 | 🗆 | 🗆 | 🗆 | 🗆 |
| 33.The needs of my patients take priority over doing hand hygiene | 🗆 | 🗆 | 🗆 | 🗆 | 🗆 |
| 34.It is important for me to help my department meet its hand hygiene goals | 🗆 | 🗆 | 🗆 | 🗆 | 🗆 |
| 35.I will feel guilty or ashamed if I omit hand hygiene | 🗆 | 🗆 | 🗆 | 🗆 | 🗆 |
| 36.I will be afraid to get infected if I omit hand hygiene | 🗆 | 🗆 | 🗆 | 🗆 | 🗆 |
| 37.I will be fear to bring home germs to anyone else if I don’t do hand hygiene | 🗆 | 🗆 | 🗆 | 🗆 | 🗆 |

**Part 3: Factors related to droplet isolation**

**Droplet isolation:** mainly including the use of personal protective equipment (PPE) (mask, glove, goggle and gown) in this survey.

**Note:** this part of the questions **does not have the correct answer**, please fill in according to your actual situation.

|  | Strongly agree | Agree | neutral | Disagree | Strongly disagree |
| --- | --- | --- | --- | --- | --- |
| 1.I know that PPE is required when contacting high-risk patients | 🗆 | 🗆 | 🗆 | 🗆 | 🗆 |
| 2.I know droplet isolation is required when in contact with high-risk patients | 🗆 | 🗆 | 🗆 | 🗆 | 🗆 |
| 3.I know how to perform droplet isolation | 🗆 | 🗆 | 🗆 | 🗆 | 🗆 |
| 4.I know how to don PPE | 🗆 | 🗆 | 🗆 | 🗆 | 🗆 |
| 5.I know how to remove PPE | 🗆 | 🗆 | 🗆 | 🗆 | 🗆 |
| 6.I am aware of evidence linking droplet isolation to healthcare-associated infections | 🗆 | 🗆 | 🗆 | 🗆 | 🗆 |
| 7.I often participate in training about droplet isolation | 🗆 | 🗆 | 🗆 | 🗆 | 🗆 |
| 8.Droplet isolation is an important part of my professional training | 🗆 | 🗆 | 🗆 | 🗆 | 🗆 |
| 9.Practicing droplet isolation is a habit of me, if necessary | 🗆 | 🗆 | 🗆 | 🗆 | 🗆 |
| 10.Easily visible posters and signs of droplet isolation remind me to practice droplet isolation | 🗆 | 🗆 | 🗆 | 🗆 | 🗆 |
| 11.I find myself forgetting to practice droplet isolation more often than others | 🗆 | 🗆 | 🗆 | 🗆 | 🗆 |
| 12.I sometimes forget to do droplet isolation because I get distracted by other things | 🗆 | 🗆 | 🗆 | 🗆 | 🗆 |
| 13.The communication with colleagues can promote me to perform droplet isolation | 🗆 | 🗆 | 🗆 | 🗆 | 🗆 |
| 14.I am often too busy to perform droplet isolation | 🗆 | 🗆 | 🗆 | 🗆 | 🗆 |
| 15. There are adequate masks and gloves | 🗆 | 🗆 | 🗆 | 🗆 | 🗆 |
| 16. There are adequate goggles and gowns | 🗆 | 🗆 | 🗆 | 🗆 | 🗆 |
| 17. I can easily access to the masks and gloves | 🗆 | 🗆 | 🗆 | 🗆 | 🗆 |
| 18. I can easily access to the goggles and gowns | 🗆 | 🗆 | 🗆 | 🗆 | 🗆 |
| 19.When busy, I am less likely to use PPE | 🗆 | 🗆 | 🗆 | 🗆 | 🗆 |
| 20.When busy, I am less likely to comply with the droplet isolation guidelines | 🗆 | 🗆 | 🗆 | 🗆 | 🗆 |
| 21.The type of my job makes it difficult to do droplet isolation | 🗆 | 🗆 | 🗆 | 🗆 | 🗆 |
| 22.My colleagues comply with the droplet isolation guidelines | 🗆 | 🗆 | 🗆 | 🗆 | 🗆 |
| 23.I comply with the droplet isolation guidelines because my colleagues do so | 🗆 | 🗆 | 🗆 | 🗆 | 🗆 |
| 24.Department leaders attach importance to droplet isolation | 🗆 | 🗆 | 🗆 | 🗆 | 🗆 |
| 25.I do droplet isolation because my patients expect me to do droplet isolation | 🗆 | 🗆 | 🗆 | 🗆 | 🗆 |
| 26.The infection management department/Infection prevention and control team urges me to do droplet isolation | 🗆 | 🗆 | 🗆 | 🗆 | 🗆 |
| 27.Staffs who do well in droplet isolation will be praised and encouraged | 🗆 | 🗆 | 🗆 | 🗆 | 🗆 |
| 28.Droplet isolation reduces/prevents the transmission of infection | 🗆 | 🗆 | 🗆 | 🗆 | 🗆 |
| 29.Droplet isolation protects me from infection | 🗆 | 🗆 | 🗆 | 🗆 | 🗆 |
| 30.Droplet isolation procedures are easy to practice | 🗆 | 🗆 | 🗆 | 🗆 | 🗆 |
| 31.I am confident that I have followed the guidelines correctly when practicing droplet isolation | 🗆 | 🗆 | 🗆 | 🗆 | 🗆 |
| 32.I do droplet isolation to protect my patients | 🗆 | 🗆 | 🗆 | 🗆 | 🗆 |
| 33. Implementing droplet isolation is my professional duty | 🗆 | 🗆 | 🗆 | 🗆 | 🗆 |
| 34.By regularly doing droplet isolation, I can be a role model for others | 🗆 | 🗆 | 🗆 | 🗆 | 🗆 |
| 35.Droplet isolation is always necessary | 🗆 | 🗆 | 🗆 | 🗆 | 🗆 |
| 36.The needs of my patients take priority over doing droplet isolation | 🗆 | 🗆 | 🗆 | 🗆 | 🗆 |
| 37.It is important for me to help the department reach its droplet isolation goals | 🗆 | 🗆 | 🗆 | 🗆 | 🗆 |
| 38.I will feel guilty or ashamed if I omit droplet isolation | 🗆 | 🗆 | 🗆 | 🗆 | 🗆 |
| 39.I will be afraid of being infected if I omit droplet isolation | 🗆 | 🗆 | 🗆 | 🗆 | 🗆 |

**Part 3: Personal characteristics**

**Note:** Your information would be kept confidential.

| **1.Gender** | □ Male | □ Female |  |  |
| --- | --- | --- | --- | --- |
| **2.Age** | years |  |  |  |
| **3.Education level** | □Associate degree or below | □Bachelor’s degree | □Master’s degree | □Doctor's degree |
| **4. Occupation** | □ Physician | □ Nurse |  |  |
| **5. Title** | □ To be appraised □ Middle □Junior □Associate Senior □Senior | | | |
| **6. Working Years** | years |  |  |  |

Investigator (name):

Date:
