# Supplementary material for: Healthcare workers’ behaviors on infection prevention and control and their determinants during the COVID-19 pandemic: a cross-sectional study based on the theoretical domains framework in Wuhan, China
Source: Arch Public Health. 2021 Jun 30;79:118. doi: 10.1186/s13690-021-00641-0 (PMC8242273; doi:10.1186/s13690-021-00641-0)
Supplement: Supplementary file 2 — Additional file 2: Table S1. TDF items for hand hygiene and their factor loadings; Table S2. TDF items for droplet isolation and their factor loadings. [file 13690_2021_641_MOESM2_ESM.docx]

**Additional file 2**

**Table S1.** TDF items for hand hygiene and their factor loadings

| Domains | No. | Items | Factor loading |
| --- | --- | --- | --- |
| Knowledge | 1 | I am aware of the five moments of hand hygiene | 0.621 |
|  | 2 | I know the content of the current hand hygiene guidelines | 0.745 |
|  | 3 | I am aware of how to perform six-step hand washing technique | 0.569 |
|  | 4 | I am aware of evidence linking hand hygiene to healthcare-associated infections | 0.504 |
| Skills | 1 | I often participate in hand hygiene training | 0.836 |
|  | 2 | Hand hygiene is an important part of my professional training | 0.836 |
| Memory, and attention | 1 | Practicing hand hygiene is a habit of me. | 0.703 |
|  | 2 | Easily visible hand hygiene stations make it easier to remember to practice hand hygiene | 0.711 |
|  | 3 | I find myself forgetting to practice hand hygiene more often than others | 0.873 |
|  | 4 | I sometimes forget to do hand hygiene because I get distracted by other things | 0.868 |
|  | *5* | *The communication with colleagues can promote me to perform hand hygiene** |  |
| Environmental context and resources | 1 | I am often too busy to do hand hygiene | 0.800 |
|  | 2 | Hand hygiene facilities are adequate | 0.802 |
|  | 3 | I can easily access to hand hygiene facilities | 0.807 |
|  | 4 | When busy, I am less likely to comply with hand hygiene guidelines | 0.821 |
|  | 5 | The type of my job makes it difficult to do hand hygiene | 0.835 |
| Social influences | 1 | My colleagues do hand hygiene as often as they should | 0.561 |
|  | 2 | Department leaders attach importance to hand hygiene | 0.681 |
|  | 3 | I do hand hygiene because my patients expect me to do hand hygiene | 0.460 |
|  | 4 | The infection management department/Infection prevention and control team urges me to do hand hygiene | 0.624 |
|  | 5 | Staffs who do well in hand hygiene will be praised and encouraged | 0.624 |
|  | *6* | *I consistently do hand hygiene because my colleagues do so** |  |
| Beliefs about consequences | 1 | Hand hygiene reduces/prevents the transmission of infection | 0.767 |
|  | 2 | Hand hygiene protects me from infection | 0.767 |
| Beliefs about capabilities | 1 | Hand hygiene procedure is easy to practice | 0.765 |
|  | 2 | I am confident in my ability to comply with hand hygiene guidelines | 0.830 |
|  | 3 | I can do hand hygiene as frequently as hand hygiene guidelines required | 0.786 |
| Social/ professional role and identity | 1 | I do hand hygiene to protect my patients | 0.855 |
|  | 2 | I do hand hygiene to protect my family | 0.514 |
|  | 3 | Hand hygiene is something I must do as part of my role | 0.823 |
|  | 4 | By regularly doing hand hygiene, I can be a role model for others | 0.736 |
| Goals | 1 | Hand hygiene is always a necessity | 0.833 |
|  | 2 | The needs of my patients take priority over doing hand hygiene | 0.670 |
|  | 3 | It is important for me to help my department meet its hand hygiene goals | 0.857 |
| Emotion | 1 | I will feel guilty or ashamed if I omit hand hygiene | 0.622 |
|  | 2 | I will be afraid to get infected if I omit hand hygiene | 0.821 |
|  | 3 | I will be fear to bring home germs to anyone else if I don’t do hand hygiene | 0.793 |

**The deleted items were marked using italic.*

**Table S2.** TDF items for droplet isolation and their factor loadings

| Domains | No. | Items | Factor loading |
| --- | --- | --- | --- |
| Knowledge | 1 | I know that PPE is required when contacting high-risk patients | 0.791 |
|  | 2 | I know droplet isolation is required when in contact with high-risk patients | 0.785 |
|  | 3 | I know how to perform droplet isolation | 0.627 |
|  | 4 | I know how to don the PPE | 0.918 |
|  | 5 | I know how to remove the PPE | 0.916 |
|  | 6 | I am aware of evidence linking droplet isolation to healthcare-associated infections | 0.493 |
| Skills | 1 | I often participate in training about droplet isolation | 0.851 |
|  | 2 | Droplet isolation is an important part of my professional training | 0.851 |
| Memory, and attention | 1 | Practicing droplet isolation is a habit of me, if necessary | 0.702 |
|  | 2 | Easily visible posters and signs of droplet isolation remind me to practice droplet isolation | 0.760 |
|  | 3 | I find myself forgetting to practice droplet isolation more often than others | 0.852 |
|  | 4 | I sometimes forget to do droplet isolation because I get distracted by other things | 0.859 |
|  | 5 | The communication with colleagues can promote me to perform droplet isolation | 0.532 |
| Environmental context and resources | 1 | I am often too busy to perform droplet isolation | 0.673 |
|  | 2 | There are adequate masks and gloves | 0.817 |
|  | 3 | There are adequate goggles and gowns | 0.826 |
|  | 4 | I can easily access to the masks and gloves | 0.805 |
|  | 5 | I can easily access to the goggles and gowns | 0.860 |
|  | 6 | When busy, I am less likely to use PPE. | 0.795 |
|  | 7 | When busy, I am less likely to comply with droplet isolation guidelines | 0.829 |
|  | 8 | The type of my job makes it difficult to do droplet isolation | 0.778 |
| Social influences | 1 | My colleagues comply with the droplet isolation guidelines | 0.612 |
|  | 2 | I comply with the droplet isolation guidelines because my colleagues do so | 0.833 |
|  | 3 | Department leaders attach importance to droplet isolation | 0.779 |
|  | 4 | I do droplet isolation because my patients expect me to do droplet isolation | 0.831 |
|  | 5 | The infection management department/Infection prevention and control team urges me to do droplet isolation | 0.623 |
|  | 6 | Staffs who do well in droplet isolation will be praised and encouraged | 0.713 |
| Beliefs about consequences | 1 | Droplet isolation reduces/prevents the transmission of infection | 0.817 |
|  | 2 | Droplet isolation protects me from infection | 0.817 |
| Beliefs about capabilities | 1 | Droplet isolation procedures are easy to practice | 0.937 |
|  | 2 | I am confident that I have followed the guidelines correctly when practicing droplet isolation | 0.937 |
| Social/ professional role and identity | 1 | I do droplet isolation to protect my patients | 0.817 |
|  | 2 | Implementing droplet isolation is my professional duty | 0.854 |
|  | 3 | By regularly doing droplet isolation, I can be a role model for others | 0.770 |
| Goals | 1 | Droplet isolation is always necessary | 0.784 |
|  | 2 | The needs of my patients take priority over doing droplet isolation | 0.703 |
|  | 3 | It is important for me to help my department reach its droplet isolation goals | 0.843 |
| Emotion | 1 | I will feel guilty or ashamed if I omit droplet isolation | 0.938 |
|  | 2 | I will be afraid of being infected if I omit droplet isolation | 0.938 |
