# Supplementary material for: Healthcare workers’ behaviors on infection prevention and control and their determinants during the COVID-19 pandemic: a cross-sectional study based on the theoretical domains framework in Wuhan, China
Source: Arch Public Health. 2021 Jun 30;79:118. doi: 10.1186/s13690-021-00641-0 (PMC8242273; doi:10.1186/s13690-021-00641-0)
Supplement: Supplementary file 3 — Additional file 3: Table S3. Robustness check. [file 13690_2021_641_MOESM3_ESM.docx]

Additional file 3

Table S3. Robustness check

|  | **Gender** | | | | **Occupation** | | | | **Age** | | | |
| --- | --- | --- | --- | --- | --- | --- | --- | --- | --- | --- | --- | --- |
|  | Female | | Male | | Nurse | | Physician | | ≥30 | | <30 | |
|  | β | *p* | β | *p* | β | *p* | β | *p* | β | *p* | β | *p* |
| **Overall hand hygiene compliance** | | | | | | | | | | | | |
| Environmental context and resources | 0.022 | 0.060 |  |  |  |  | 0.048 | 0.008 | 0.045 | <0.001 |  |  |
| **Compliance of overall droplet isolation behaviors** | | | | | | | | | | | | |
| Knowledge | 0.086 | 0.024 |  |  | 0.110 | 0.013 |  |  |  |  | 0.082 | 0.060 |
| Environmental context and resources | 0.083 | 0.002 |  |  | 0.078 | 0.005 |  |  | 0.119 | <0.001 |  |  |
| Social influence | 0.047 | 0.073 |  |  | 0.048 | 0.073 |  |  |  |  | 0.086 | 0.012 |
| Emotion | 0.078 | 0.032 |  |  | 0.077 | 0.044 |  |  | 0.077 | 0.072 | 0.113 | 0.025 |
| **Use of goggle** | | | | | | | | | | | | |
| Contact with confirmed or suspected patients | -0.127 | 0.082 |  |  | -0.171 | 0.022 |  |  |  |  | -0.199 | 0.050 |
| Knowledge | 0.237 | 0.001 |  |  | 0.201 | 0.013 |  |  | 0.185 | 0.029 | 0.184 | 0.041 |
| Environmental context and resources | 0.133 | 0.005 |  |  | 0.127 | 0.008 | 0.136 | 0.076 | 0.216 | <0.001 |  |  |
| Social influence |  |  |  |  |  |  |  |  |  |  | 0.133 | 0.045 |
| Emotion | 0.135 | 0.050 |  |  | 0.133 | 0.063 |  |  | 0.152 | 0.037 |  |  |
| Education degree |  |  | -0.138 | 0.030 |  |  |  |  | -0.098 | 0.087 |  |  |
| **Use of gown** | | | | | | | | | | | | |
| Contact with confirmed or suspected patients | -0.165 | 0.003 |  |  | -0.203 | <0.001 |  |  |  |  | -0.237 | 0.001 |
| Knowledge | 0.142 | 0.007 |  |  | 0.159 | 0.008 |  |  | 0.127 | 0.083 | 0.124 | 0.045 |
| Environmental context and resources | 0.142 | <0.001 | 0.124 | 0.047 | 0.129 | <0.001 | 0.160 | 0.010 | 0.204 | <0.001 |  |  |
| Social/professional role and identity |  |  |  |  | 0.058 | 0.082 | -0.297 | 0.032 | -0.186 | 0.088 |  |  |
| Emotion | 0.220 | <0.001 |  |  | 0.229 | <0.001 | 0.152 | 0.093 | 0.208 | 0.001 | 0.199 | 0.008 |
| Education degree |  |  | -0.104 | 0.050 |  |  | -0.084 | 0.080 | -0.093 | 0.057 |  |  |
